# A literature review of the healthcare resource use and productivity burden of X-linked hypophosphataemia

**DOI:** 10.3389/frhs.2025.1285246

**Published:** 2025-04-09

**Authors:** Rafael Pinedo-Villanueva, Muhammad K. Javaid, Angela Williams, Isabelle Whittle, Matilde Franceschini, Ben Johnson

**Affiliations:** ^1^ Nuffield Department of Orthopaedics, Rheumatology and Musculoskeletal Sciences, University of Oxford, Oxford, United Kingdom; ^2^ Department of Health Economics and Outcomes Research, Kyowa Kirin International, Marlow, United Kingdom; ^3^ Department of Value Insight and Communication, Adelphi Values PROVE, Bollington, United Kingdom

**Keywords:** X-linked hypophosphataemia, healthcare resource use, rare diseases, productivity loss, economic burden

## Abstract

**Introduction:**

X-linked hypophosphataemia (XLH) is a rare, genetic, renal phosphate wasting disorder that causes a lifelong rapid progression of morbidities, which are associated with substantial humanistic and economic burden. A structured literature review was carried out to identify publications reporting healthcare resource use and productivity impact of XLH to provide a comprehensive description of the burden.

**Methods:**

Literature searches of the Embase®, Medline®, and EconLit electronic databases were carried out in August 2022 using free-text and subject heading search terms regarding XLH-related clinical morbidities and associated healthcare resource use, limited to English language records from 1992 onwards.

**Results:**

After screening by pre-specified inclusion/exclusion criteria, 22 publications were selected for inclusion in the review. Use of conventional pharmacological therapy with oral phosphate and/or active vitamin D was reported in 15 publications, in up to 100% of paediatric patients and 75% of adults. Findings indicated that a high proportion of patients with XLH undergo orthopaedic procedures/surgeries, including a history of osteotomy in up to 25% of paediatric patients and 61% of adults, and a history of growth plate stapling in up to 63% of paediatric patients and 20% of adults. A high prevalence of fractures (in up to 61% of adults) and use of assistive mobility devices was also reported. The findings highlighted a substantial prevalence of morbidities, either due to persistently low phosphate levels or complications of conventional therapy, that had directly associated healthcare resource use, including dental problems, hearing problems, hyperparathyroidism, and nephrocalcinosis. Healthcare resource use and associated clinical events were generally found to be higher in adults compared with paediatric patients, which is consistent with the natural history of XLH as a progressive lifelong condition. Studies also highlighted the negative impact of XLH on school attendance and the ability to work.

**Discussion:**

The results of this structured literature review emphasise the lifelong impact of XLH, showing that it is associated with a substantial economic burden, across many healthcare resource use categories including pharmacological therapy, management of pain and mobility, orthopaedic procedures, morbidities due to XLH or conventional therapy, and work/school productivity.

## 1 Introduction

X-linked hypophosphataemia (XLH) is a rare, genetic, progressive, and lifelong renal phosphate wasting disorder caused by loss-of-function mutations in the *PHEX* (phosphate-regulating endopeptidase homologue, X-linked) gene that results in excess circulating levels of fibroblast growth factor 23 (FGF23) (1–4). The population prevalence of XLH is estimated to be between approximately 1 in 20,000 and 1 in 60,000, and approximately 20%–30% of cases are spontaneous (5–10). Clinical manifestations of XLH usually begin in early childhood and include rickets and weakened skeletal bones that result in lower limb deformities and shortened stature (11). The presence of these deformities, ongoing chronic hypophosphataemia, and complications of conventional phosphate and active vitamin D therapy lead to the development of further debilitating morbidities in adulthood, including fractures, enthesopathy, spinal stenosis, and osteoarthritis as well as nephrocalcinosis and hyperparathyroidism (12–15).

Skeletal disease frequently leads to the requirement for orthopaedic procedures, such as osteotomy, stapling of growth plates, hip and knee replacement, and spinal surgery (12, 13, 15, 16). Most patients experience pain, stiffness, and fatigue that can have a considerable impact on their mobility and their ability to perform daily activities, as well as limiting their social, family, and work life (12, 17, 18). Patients with XLH also present with dental problems throughout life, owing to defects in dentin and enamel (12, 13), and premature hearing loss.

Pharmacological treatment of XLH has traditionally been based on supplementation with oral phosphate and active vitamin D (“conventional therapy”) (11, 12, 14, 19). However, this regimen requires administration several times a day, generally produces only a transient improvement but not normalisation of serum phosphate levels, and is associated with a number of side effects including gastrointestinal events, nephrocalcinosis, kidney stones, and hyperparathyroidism (1, 11, 12). Treatment with conventional therapy is typically recommended in all children but is only recommended in adults who are symptomatic due to adverse effects and the burden of administration (14, 19).

As an alternative to conventional therapy, burosumab is a fully human immunoglobulin G1 (IgG1) monoclonal antibody which inhibits FGF23 activity and therefore directly addresses the pathological mechanism of hypophosphataemia in XLH (20). Burosumab has been approved for the treatment of XLH in a wide range of markets globally including in the United States, European Union, and Canada in 2018, and in Japan in 2019 (21–24), and has shown efficacy in normalising serum phosphate levels.

XLH can be associated with a substantial economic burden across multiple categories, including use of pharmacological treatments, routine disease and treatment monitoring, orthopaedic interventions, and loss of productivity (12–16, 19, 25). The aim of this study is to conduct a structured literature review to identify and provide a comprehensive description of the healthcare resource use and productivity burden associated with XLH over a lifetime.

## 2 Methods

### 2.1 Scope of the review

A structured literature review was conducted to identify publications containing information on clinical morbidities, associated healthcare resource use, and productivity loss due to XLH. Clinical events known to be associated with healthcare resource use were included in the scope of the review, including fractures, dental problems, and hyperparathyroidism, as well as direct reporting of healthcare resource use to ensure a comprehensive review.

### 2.2 Literature search strategy

Literature searches of the Embase®, Medline®, and EconLit (published by the American Economic Association) electronic databases were carried out via Ovid SP® on 16 August 2022, using free-text and subject heading search terms.

The searches were limited to English language records between 1992 and the present day (i.e., a publication range of 30 years) without any geographical restrictions. This date range was selected to encompass a broad range of publications, while remaining relevant to current practice.

The full search strategies are provided in Supplementary File S1, Tables 1–3 for the Embase®, Medline®, and EconLit searches, respectively.

### 2.3 Validation

The search strategies were validated by checking that the obtained results included six key XLH control publications (12–16, 19), all of which were successfully identified.

### 2.4 Screening

Titles and abstracts of identified publications were screened for eligibility using the inclusion and exclusion criteria defined in Supplementary File S2, Table 1. Only full text publications were included in the review; conference abstracts were excluded. Small studies, with a population size of less than 10 patients, were also excluded. Full text articles which were not excluded at the title/abstract screening stage were further reviewed against the inclusion and exclusion criteria. At both stages, screening was conducted by one reviewer and quality checked by a second reviewer.

### 2.5 Data extraction

Data from identified full text publications were extracted into a Microsoft Excel document. The following information was recorded for each study: publication (authors, year, title), study design (study type, country, data collection period), patient population (characteristics, sample size, age range), and healthcare resource use/clinical event outcomes (resource use category, statistical measure, and estimate). If possible, outcomes were extracted separately for paediatric and adult patients.

In addition to excluding studies with a total sample size <10 patients, any specific outcomes with a sample size <10 were also excluded. In order to avoid introducing bias and therefore maintain generalisability, in studies where the patient population was selected on the basis of a particular event associated with healthcare resource use, any related outcomes were excluded. For example, if a study was conducted in a sample of patients who had undergone parathyroidectomy, data on oral phosphate/active vitamin D use, hyperparathyroidism, and parathyroidectomy would not be extracted. Furthermore, it was decided to exclude burosumab from the review as it has only recently become available (in 2018/2019) and therefore its use is not consistently established, and it is unlikely that the potential impact of burosumab on resource use would have been adequately captured. In addition, longer-term outcomes (such as orthopaedic interventions, morbidities, etc.) would reflect the treatment landscape prior to the availability of burosumab.

Healthcare resource use outcomes were grouped into the following categories:

• Pharmacological therapy and surveillance/treatment monitoring of XLH: pharmacological treatment and routine monitoring of disease/treatment (e.g., laboratory tests and healthcare appointments)
• Orthopaedic interventions: procedures to correct skeletal deformities associated with XLH, events which require orthopaedic intervention (fractures), and mobility device use
• Treatment of other morbidities related to XLH: management of outcomes associated with chronic hypophosphataemia (e.g., dental and hearing problems)
• Treatment of morbidities related to conventional therapy: management of adverse effects of long-term treatment with oral phosphate and active vitamin D
• Productivity loss: absenteeism and presenteeism at work or school due to the disease

## 3 Results

### 3.1 Results of the literature searches

**Figure 1:**
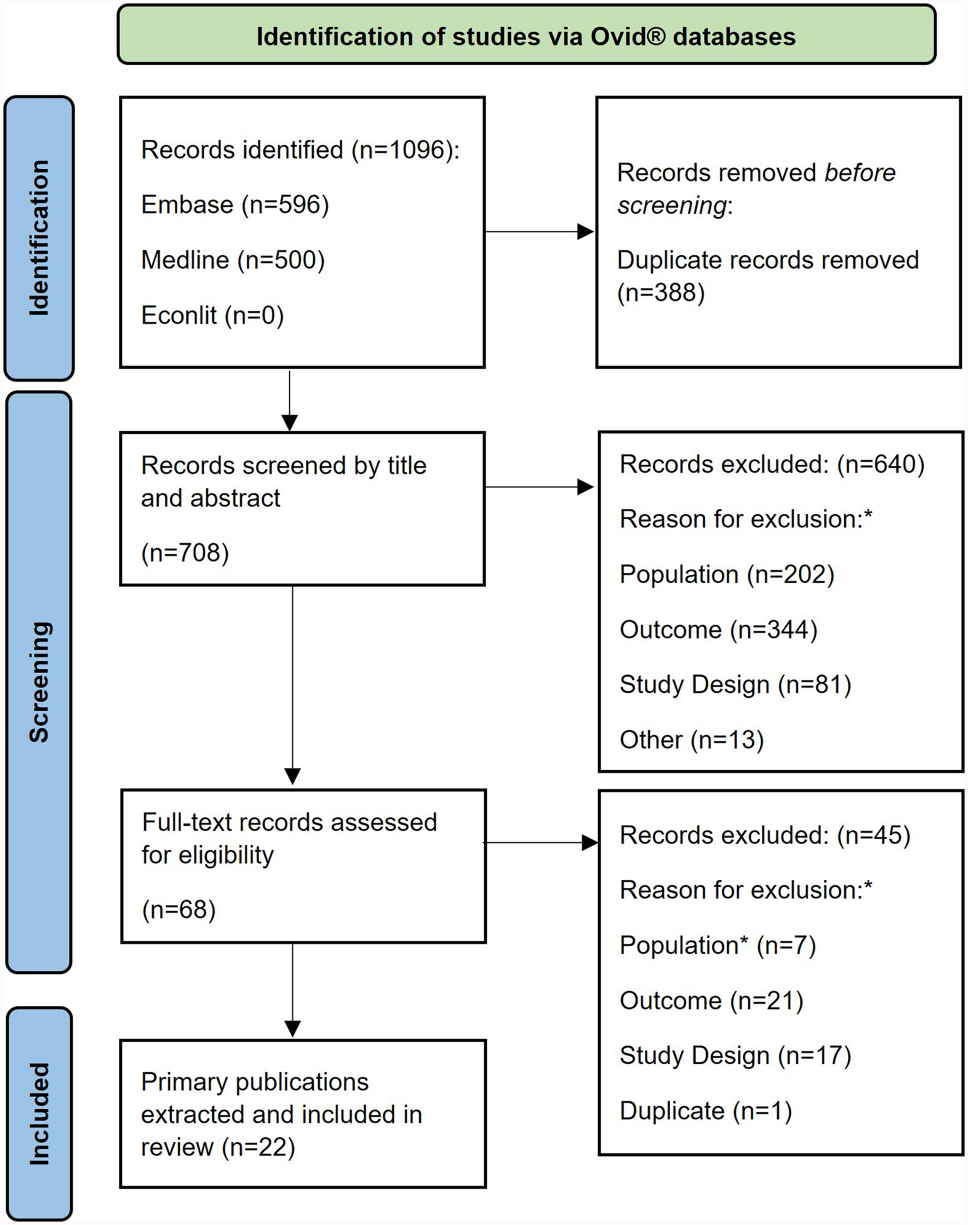
PRISMA flow diagram. *Study inclusion and exclusion criteria are described in full in (Supplementary File S2).

Figure 1 shows the PRISMA diagram for the selection of eligible publications. After removing any duplicate references, a total of 708 primary publications (titles/abstracts) were identified in the electronic databases, 68 of which were selected for full text screening. After screening, 22 publications were included in the review.

A summary of the key characteristics of the included studies is provided in Table 1. The most commonly reported designs were retrospective study (*n* = 11), followed by cross-sectional study (*n* = 6), and observational research (*n* = 2). The most common geographical locations for the studies were France (*n* = 4) and the United States (*n* = 3).

**Table 1:** Characteristics of the studies included in the literature review (*N* = 22).

Author, year	Study design^a^	Country	Study period (duration)	Data source	Patient type (sample size)	Patient age	XLH diagnosis criteria
Alon et al. (2003) (26)	Retrospective study	USA; Netherlands	NR	Medical records	Paediatric (*n* = 41)	Range: 10–18 years	NR
Baroncelli et al. (2021) (27)	Longitudinal study	Italy	NR	Medical records	Adult and paediatric (*n* = 26)	Range: 3.1–25.7 years	NR
Berndt et al. (1996) (28)	Retrospective study	Germany	1940–1991 (51 years)	Clinical data	Adult (*n* = 23)	Median: 11 years	Reduced tubular phosphate reabsorption, persistent hypophosphatemia, history of rickets
Chesher et al. (2018) (13)	Case-note review	UK	1998	Clinical records	Adult (*n* = 59)	Median: 37 years	NR (*PHEX* gene mutation in 48 patients, genotype inferred from relative in 11)
Chung et al. (2002) (29)	Retrospective study	Taiwan	1983–2001 (18 years)	Medical records	Adult and paediatric (*n* = 15)	Median: 5.6 years	NR
DeLacey et al. (2019) (30)	Retrospective study	USA	2000–2017 (17 years)	Medical records	Adult and paediatric (*n* = 84)	Age groups: ≥18 years, *n* = 40 <18 years, *n* = 44	Hypophosphatemia with hyperphosphaturia beginning in childhood, clinical history consistent with XLH
Fucentese et al. (2008) (31)	Retrospective study	Switzerland	1976–NR	Medical records	Paediatric (*n* = 12)	Median: 13 years	NR
Gizard et al. (2017) (32)	Retrospective study	France	1974–2014 (40 years)	Medical records	Paediatric (*n* = 49)	Mean: 6 years	Clinical and biochemical profile consistent with XLH
Herrou et al. (2022) (33)	Retrospective study	France	2011–2020 (9 years)	Medical records	Adult (*n* = 114)	Mean (SD): 42.2 (14.3) years	*PHEX* mutation, or variant of unknown significance in patient/relative with X-linked dominant inheritance, or hypophosphatemia with excess FGF23
Horn et al. (2017) (34)	Retrospective study	NR	NR	Medical records and radiographs	Paediatric (*n* = 24)	Mean: 1.5 years	NR
Ito et al. (2022) (16)	Cross-sectional study	Japan; Korea	2017–2019 (2 years)	Survey responses	Adult and paediatric (*n* = 46)	Mean (SD): <18 years, 6.8 (3.7) years ≥18 years, 40.2 (13.8) years	Survey respondent-reported diagnosis
Javaid et al. (2022) (15)	Cross-sectional study	Multinational	2014–2016 (2 years)	Clinical trial survey	Adult (*n* = 336)	Clinical trial group mean (SD): 39.8 (12.3) years Survey group mean (SD): 45.5 (13.0) years	Clinical trial group: clinical and biochemical features of XLH and/or confirmed *PHEX* mutation in patient/family member Survey group: survey respondent-reported diagnosis
Jiménez et al. (2021) (35)	Cross-sectional study	Chile	NR	Medical records	Adult and paediatric (*n* = 26)	Mean (SD): 29.5 (16.4) years	Clinical, radiological, and biochemical findings indicating congenital hypophosphataemia associated with renal phosphate wasting
Lecoq et al. (2020) (36)	Observational	France	2011–2017 (6 years)	Clinical data	Adult (*n* = 68)	Median: 38 years	Hypophosphatemia due to renal phosphate wasting plus *PHEX* mutation and/or family history of rickets and/or increased FGF23 concentration
Moreira et al. (2020) (37)	Cross-sectional study	Brazil	2018	Survey responses	Adult and paediatric (*n* = 57)	Median: 22 years	Clinical and laboratory features of XLH and/or *PHEX* gene mutation
Rafaelsen et al. (2016) (6)	Observational	Norway	2009	Clinical data	Adult (*n* = 21)	Median: 22 years	*PHEX* gene mutation
Rodríguez-Rubio et al. (2021) (38)	Retrospective study	Spain	NR	Medical records	Paediatric (*n* = 48)	Median (IQR): 2.0 (2.6) years	*PHEX* gene mutation
Rohmiller et al. (1999) (39)	Retrospective study	USA	1975–1996 (21 years)	Medical records	Paediatric (*n* = 38)	Range: 3–213 months	Lower extremity deformities, radiographic evidence of rickets, and biochemical features
Rothenbuhler et al. (2019) (40)	Retrospective study	France	2001–2015 (14 years)	Medical records	Paediatric (*n* = 44)	Mean (SD): 8.7 (3.9) years	Biochemical features and disease transmission (*PHEX* mutation confirmed in 31/44 patients)
Sant’ Ana et al. (2022) (41)	Cross-sectional study	Brazil	NR	Medical records	Adults and paediatric (*n* = 16)	Range: 5–38 years	*PHEX* gene mutation
Skrinar et al. (2019) (12)	Cross-sectional study	Multinational (>30 countries, including USA)	2014–2016 (2 years)	Patient survey	Adults and paediatric (*n* = 322)	Adult mean (SD): 45.6 (12.9) years Paediatric mean (SD): 9.1 (3.9) years	Survey respondent-reported diagnosis
Theodore-Oklota et al. (2018) (42)	Qualitative interview	USA	NR	Patient reported perspective	Adult (*n* = 18)	Mean: 42 years	NR

Abbreviations: IQR, interquartile range; NR, not reported; SD, standard deviation; UK, United Kingdom; USA, United States of America; XLH, X-linked hypophosphataemia.

^a^ Study type as reported by the authors.

Seven studies reported outcomes for adult patients, the same number reported outcomes in paediatric patients, and eight studies included both adults and paediatric patients. Most of the included publications (*n* = 17) were published in the last 10 years (i.e., with a publication date from 2012 onwards).

### 3.2 Healthcare resource use findings

#### 3.2.1 Pharmacological therapy and surveillance/treatment monitoring of XLH

**Figure 2:**
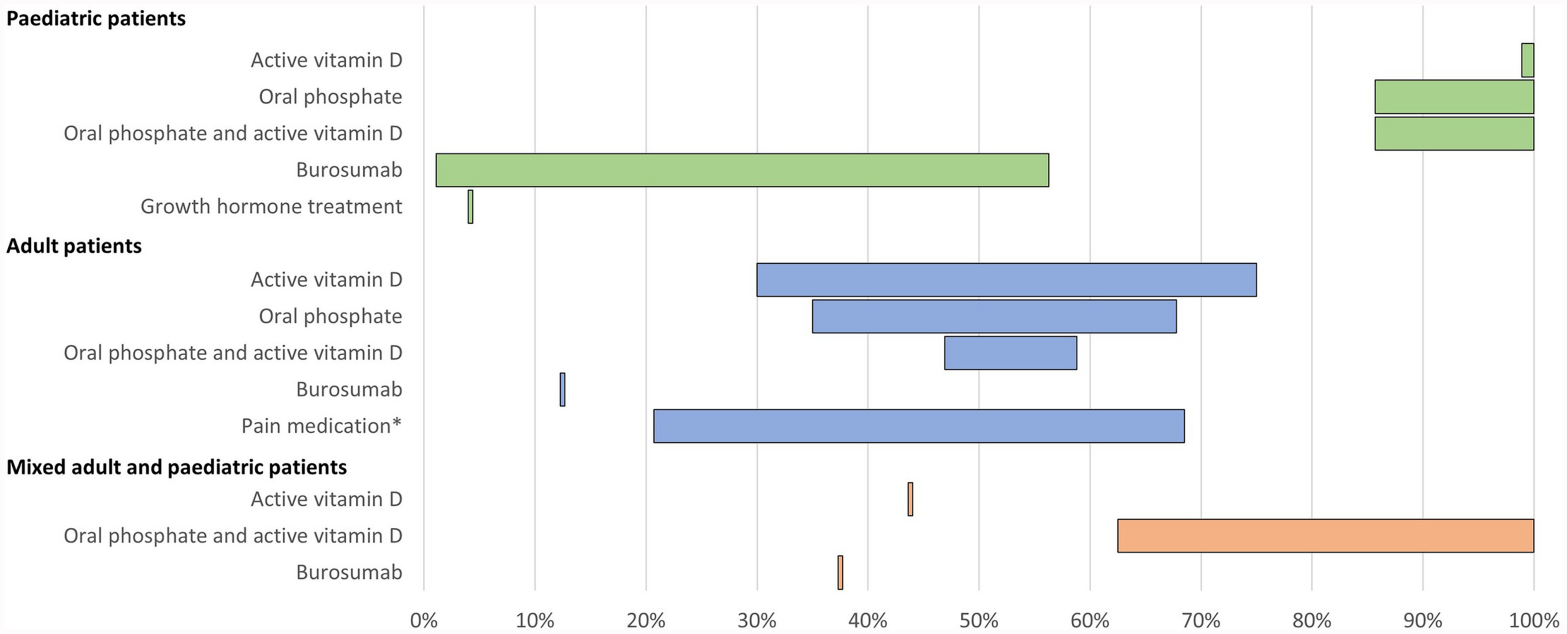
Range of the proportion of patients treated with pharmacological therapy reported across studies. *Including over-the-counter pain medication and/or prescription pain medication. Abbreviations: XLH, X-linked hypophosphataemia.

A total of 15 publications reported use of pharmacological treatments for XLH (Table 2; Figure 2).

**Table 2:** Summary of pharmacological therapy and surveillance/treatment monitoring of XLH.

Treatment	Publication	Sample size (*n*)	Number of patients (%)	Age
Paediatric patients				
Active vitamin D	Fucentese et al. (2008) (31)	12	12 (100)	Median: 13 years
	Ito et al. (2022) (16)	48	48 (100)	Median (IQR): 2.0 (2.6) years
	Skrinar et al. (2019) (12)	90	89 (98.9)	Mean (SD): 9.1 (3.9) years
Oral phosphate	Fucentese et al. (2008) (31)	12	12 (100)	Median: 13 years
	Ito et al. (2022) (16)	14	12 (85.7)	Mean (SD): 6.8 (3.7) years
	Skrinar et al. (2019) (12)	90	89 (98.9)	Mean (SD): 9.1 (3.9) years
	Rodríguez-Rubio et al. (2021) (38)	48	48 (100)	Median (IQR): 2.0 (2.6) years
Oral phosphate and active vitamin D	Alon et al. (2003) (26)	41	41 (100)	Range: 10–18 years
	Gizard et al. (2017) (32)	49	42 (85.7)	Mean: 6 years
	Skrinar et al. (2019) (12)	90	89 (98.9)	Mean (SD): 9.1 (3.9) years
	Rodríguez-Rubio et al. (2021) (38)	48	48 (100)	Range: 3–213 months
Growth hormone treatment	Rodríguez-Rubio et al. (2021) (38)	48	2 (4.2)	Median (IQR): 2.0 (2.6) years
Adult patients				
Active vitamin D	Chesher et al. (2018) (13)	59	40 (67.8)	Median: 37 years
	Herrou et al. (2022) (33)	114	67 (58.8)	Mean (SD): 42.2 (14.3) years
	Ito et al. (2022) (16)	32	24 (75.0)	Mean (SD): 40.2 (13.8) years
	Jiménez et al. (2021) (35)	20	6 (30.0)	Mean (SD): 35.6 (13.4) years
	Lecoq et al. (2020) (36)	68	35 (51.5)	Median: 38 years
	Skrinar et al. (2019) (12)	232	149 (64.2)	Mean (SD): 45.6 (12.9) years
	Theodore-Oklota et al. (2018) (42)	18	10 (55.0)	Mean: 42 years
Oral phosphate	Chesher et al. (2018) (13)	59	40 (67.8)	Median: 37 years
	Ito et al. (2022) (16)	32	20 (62.5)	Mean (SD): 40.2 (13.8) years
	Lecoq et al. (2020) (36)	68	31 (45.6)	Median: 38 years
	Skrinar et al. (2019) (12)	232	114 (49.1)	Mean (SD): 45.6 (12.9) years
	Herrou et al. (2022) (33)	114	67 (58.8)	Mean (SD): 42.2 (14.3) years
	Jiménez et al. (2021) (35)	20	7 (35.0)	Mean (SD): 35.6 (13.4) years
	Theodore-Oklota et al. (2018) (42)	18	11 (61.1)	Mean: 42 years
Oral phosphate and active vitamin D	Herrou et al. (2022) (33)	114	67 (58.8)	Mean (SD): 42.2 (14.3) years
	Javaid et al. (2022) (15)^a^	127	69 (54.3)	Range: 18–65 years
		209	98 (46.9)	
	Skrinar et al. (2019) (12)	232	110 (47.4)	Mean (SD): 45.6 (12.9) years
Over-the-counter pain medication use	Skrinar et al. (2019) (12)	232	159 (68.5)	Mean (SD): 45.6 years (12.9)
Prescription pain medication use	Skrinar et al. (2019) (12)	232	48 (20.7)	Mean (SD): 45.6 years (12.9)
Use of pain medication	Jiménez et al. (2021) (35)	17	9 (52.9)	Mean (SD): 35.6 years (13.4)
Mixed adult and paediatric patients				
Active vitamin D	Sant Ana et al. (2022) (41)	16	7 (43.8)	Range: 5–38 years
Oral phosphate and active vitamin D	Sant Ana et al. (2022) (41)	16	10 (62.5)	Range: 5–38 years
	Chung et al. (2002) (29)	15	15 (100)	Median: 5.6 years, range: 1.5–24 years
	Baroncelli et al. (2021) (27)	26	17 (65.4)	Range: 3.1–25.7 years

Abbreviations: IQR, interquartile range; SD, standard deviation; XLH, X-linked hypophosphataemia.

^a^ Javaid et al. (15), reports outcomes for two groups of patients: subjects of a clinical trial and participants in an online survey.

All 15 studies reported use of conventional therapy, i.e., oral phosphate and/or active vitamin D. In paediatric patients, treatment with oral phosphate and active vitamin D was reported in 86%–100% of patients (*n* = 228 across four studies (12, 26, 32, 38), oral phosphate treatment was reported in 86%–100% of patients (*n* = 164 across four studies (12, 16, 31, 38), and active vitamin D treatment was reported in 99%–100% of patients (*n* = 150 across three studies (12, 16, 31). In adults, oral phosphate and active vitamin D treatment was reported in 47%–59% of patients (*n* = 682 across three studies (12, 15, 33), oral phosphate treatment was reported in 35%–68% of patients (*n* = 543 across seven studies (12, 13, 16, 33, 35, 36, 42), and active vitamin D treatment was reported in 30%–75% of patients (*n* = 543 across seven studies (12, 13, 16, 33, 35, 36, 42).

Additionally, one study reported use of growth hormone in 4% of paediatric patients (*n* = 48) (38). In one study of 232 adult patients, the use of over-the-counter pain medication and the use of prescription pain medication for XLH was reported by 69% and 21% of patients, respectively (12). Use of any form of pain medication was reported by 53% of adults in another study (*n* = 17) (35).

No empirical data were found on treatment surveillance and/or monitoring.

#### 3.2.2 Orthopaedic interventions

**Figure 3:**
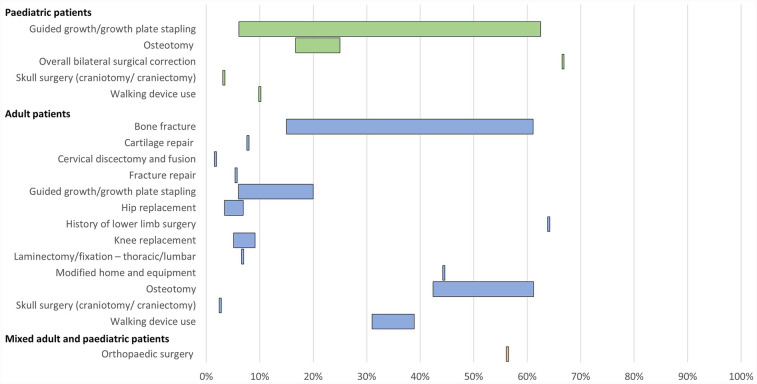
Range of the proportion of patients with a history of orthopaedic interventions associated with XLH reported across studies. Abbreviations: XLH, X-linked hypophosphataemia.

10 publications reported orthopaedic interventions associated with XLH (Table 3; Figure 3). This comprised a wide range of procedures, including osteotomy, guided growth/growth plate stapling, hip and knee replacement/arthroplasty, cartilage repair, cervical discectomy and fusion, and laminectomy.

**Table 3:** Summary of orthopaedic interventions associated with XLH.

Outcome	Publication	Sample size (*n*)	Number of patients (%)	Patient age
Paediatric patients				
Guided growth/growth plate stapling	Gizard et al. (2017) (32)	49	3 (6.1%)	Mean: 6 years
	Skrinar et al. (2019) (12)	90	12 (13.3%)	Mean (SD): 9.1 (3.9) years
	Horn et al. (2017) (34)	24	15 (62.5%)	Mean: 1.5 years†
Osteotomy	Fucentese et al. (2008) (31)	12	3 (25.0%)	Median: 13 years
	Skrinar et al. (2019) (12)	90	15 (16.7%)	Mean (SD): 9.1 (3.9) years
Overall bilateral surgical correction	Fucentese et al. (2008) (31)	12	8 (66.7%)	Median: 13 years
Skull surgery (craniotomy craniectomy)	Skrinar et al. (2019) (12)	90	3 (3.3%)	Mean (SD): 9.1 (3.9) years
Walking device use	Skrinar et al. (2019) (12)	90	9 (10.0%)	Mean (SD): 9.1 years (3.9)
Adult patients				
Bone fracture	Berndt et al. (1996) (28)	23	9 (39.1%)	Median: 29 years
	Theodore-Oklota et al. (2018) (42)	18	11 (61.1%)	Mean: 42 years
	Ito et al. (2022) (16)	32	11 (34.4%)	Mean (SD): 40.2 (13.8) years
	Javaid et al. (2022) (15)^b^	127	55 (43.3%)	Range: 18–65 years
		209	91 (43.5%)	
	Jiménez et al. (2021) (35)	20	3 (15.0%)	Mean (SD): 35.6 (13.4) years
	Skrinar et al. (2019) (12)	232	102 (44.0%)	Mean (SD): 45.6 (12.9) years
Cartilage repair	Skrinar et al. (2019) (12)	232	18 (7.8%)	Mean (SD): 45.6 (12.9) years
Cervical discectomy and fusion	Chesher et al. (2018) (13)	59	1 (1.7%)	Median: 37 years
Fracture repair	Skrinar et al. (2019) (12)	232	13 (5.6%)	Mean (SD): 45.6 (12.9) years
Guided growth/growth plate stapling	Chesher et al. (2018) (13)	59	4 (6.8%)	Median: 37 years
	Jiménez et al. (2021) (35)	20	4 (20.0%)	Mean (SD): 35.6 (13.4) years
	Skrinar et al. (2019) (12)	232	14 (6.0%)	Mean (SD): 45.6 (12.9) years
Hip replacement	Chesher et al. (2018) (13)	59	2 (3.4%)	Median: 37 years
	Javaid et al. (2022) (15)^a^	127	7 (5.5%)	Range: 18–65 years
		209	14 (6.7%)	
	Skrinar et al. (2019) (12)	232	16 (6.9%)	Mean (SD): 45.6 (12.9) years
History of lower limb surgery	Herrou et al. (2022) (33	114	73 (64.0%)	Mean (SD): 42.2 (14.3) years
Knee replacement	Chesher et al. (2018) (13)	59	3 (5.1%)	Median: 37 years
	Javaid et al. (2022) (15)^a^	127	7 (5.5%)	Range: 18–65 years
		209	17 (8.1%)	
	Skrinar et al. (2019) (12)	232	21 (9.1%)	Mean (SD): 45.6 (12.9) years
Laminectomy/fixation—thoracic/lumbar	Chesher et al. (2018) (13)	59	4 (6.8%)	Median: 37 years
Modified home and equipment	Theodore-Oklota et al. (2018) (42)	18	8 (44.4%)	Mean: 42 years
Osteotomy	Chesher et al. (2018) (13)	59	25 (42.4%)	Median: 37 years
	Jiménez et al. (2021) (35)	20	10 (50.0%)	Mean (SD): 35.6 (13.4) years
	Skrinar et al. (2019) (12)	232	142 (61.2%)	Mean (SD): 45.6 (12.9) years
Skull surgery (craniotomy craniectomy)	Skrinar et al. (2019) (12)	232	6 (2.6%)	Mean (SD): 45.6 (12.9) years
Walking device use	Skrinar et al. (2019) (12)	232	72 (31.0%)	Mean (SD): 45.6 years (12.9)
	Theodore-Oklota et al. (2018) (42)	18	7 (38.9%)	Mean: 42 years
Mixed adult and paediatric patients				
Orthopaedic surgery	Sant Ana et al. (2022) (41)	16	9 (56.3%)	Range: 5–38 years

Abbreviations: SD, standard deviation; XLH, X-linked hypophosphataemia.

^a^ Javaid et al. (15), reports outcomes for two groups of patients: subjects from a clinical trial and participants in an online survey. Only the proportion, rather than the absolute number, of patients with each event was reported. Therefore, outcomes are recorded as the range of proportions across the two groups.

^b^ Mean age at treatment initiation.

In paediatric patients, a history of osteotomy was reported in 17%–25% of patients (*n* = 102 in two studies (12, 31) and a history of guided growth/growth plate stapling was reported in 6%–63% of patients (*n* = 163 in three studies (12, 32, 34). In adults, a history of osteotomy was reported in 42%–61% of patients and a history of guided growth/growth plate stapling was reported in 6%–20% of patients (*n* = 311 across three studies (12, 13, 35).

History of hip replacement/arthroplasty was reported in 3%–7% of adult patients and knee replacement/arthroplasty was reported in 5%–9% of adults (*n* = 627 across three studies (12, 13, 15). Spinal laminectomy or fixation was reported in 7% of adults (*n* = 59 in one study (13).

A history of bone fracture was reported in 15%–61% of adult patients (*n* = 661 across six studies (12, 15, 16, 28, 35, 42). The use of assistive walking devices was reported in 10% of paediatric patients (*n* = 90 in one study (12) and 31%–39% of adult patients (*n* = 250 across two studies (12, 42). Furthermore, one publication reported that 44% of adult patients with XLH required modified home and equipment (*n* = 18) (42).

#### 3.2.3 Morbidities associated with XLH

**Figure 4:**
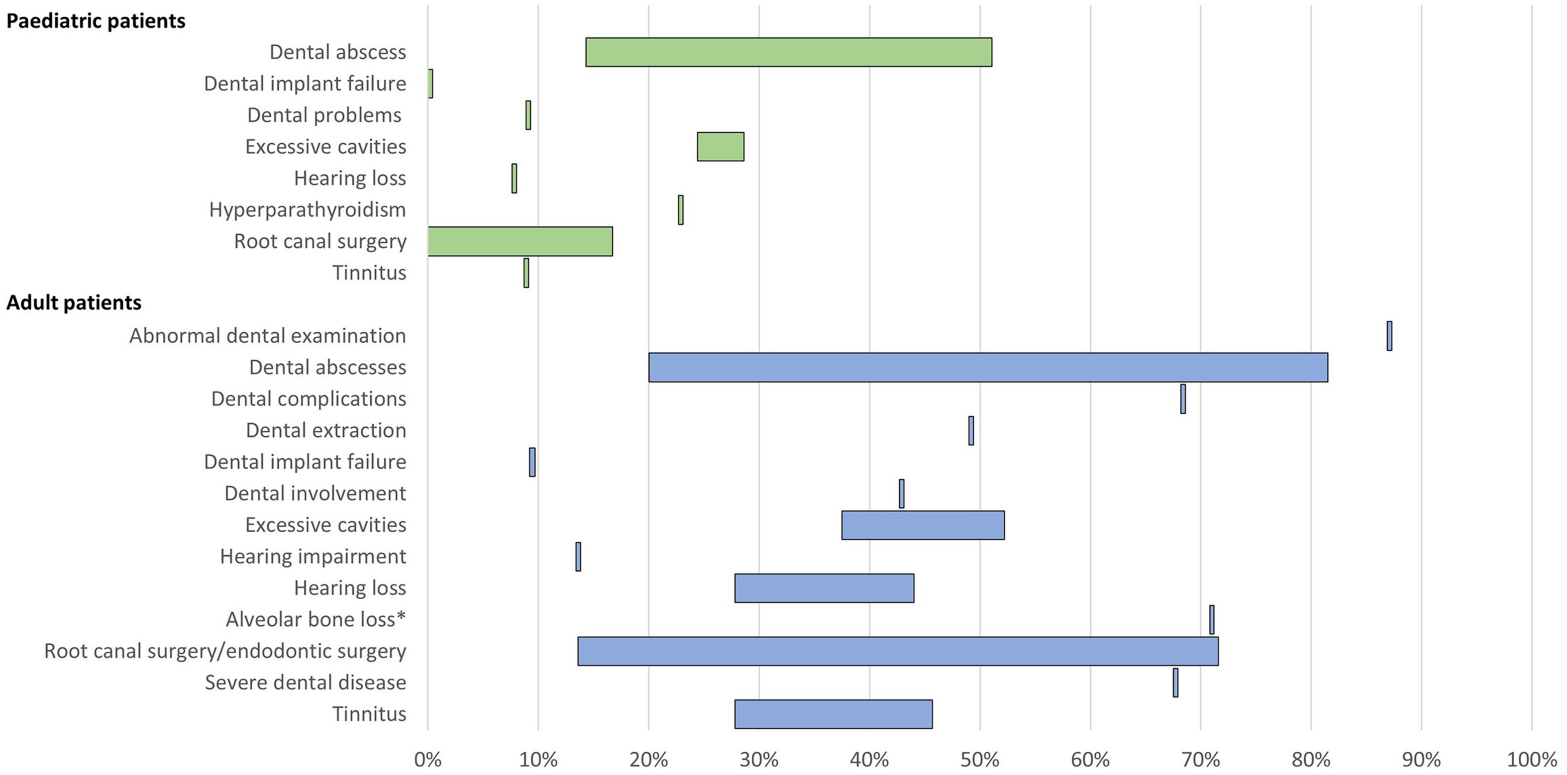
Range of the proportion of patients with a history of morbidities related to low phosphate levels of XLH reported across studies. *Includes patients with mild, moderate, and severe alveolar bone loss. Abbreviations: XLH, X-linked hypophosphataemia.

12 publications reported clinical morbidities and healthcare resource use associated with these morbidities (Table 4; Figure 4).

**Table 4:** Summary of morbidities related to low phosphate levels of XLH.

Outcome	Publication	Sample size (*n*)	Number of patients (%)	Patient age
Paediatric patients				
Dental abscess	Ito et al. (2022) (16)	14	2 (14.3%)	Mean (SD): 6.8 (3.7) years
	Skrinar et al. (2019) (12)	90	46 (51.1%)	Mean (SD): 9.1 (3.9) years
	Rothenbuhler et al. (2019) (40)	44	20 (45.5%)	Mean (SD): 8.7 (3.9) years
Dental implant failure	Ito et al. (2022) (16)	14	0 (0.0%)	Mean (SD): 6.8 (3.7) years
Dental problems	Rodríguez-Rubio et al. (2021) (38)	33	3 (9.1%)	Median (IQR): 2.0 (2.6) years
Excessive cavities	Ito et al. (2022) (16)	14	4 (28.6%)	Mean (SD): 6.8 (3.7) years
	Skrinar et al. (2019) (12)	90	22 (24.4%)	Mean (SD): 9.1 (3.9) years
Hearing loss	Skrinar et al. (2019) (12)	90	7 (7.8%)	Mean (SD): 9.1 (3.9) years
Hyperparathyroidism	Rodríguez-Rubio et al. (2021) (38)	48	11 (22.9%)	Median (IQR): 2.0 (2.6) years
Root canal surgery	Ito et al. (2022) (16)	14	0 (0.0%)	Mean (SD): 6.8 (3.7) years
	Skrinar et al. (2019) (12)	90	15 (16.7%)	Mean (SD): 9.1 (3.9) years
Tinnitus	Skrinar et al. (2019) (12)	90	8 (8.9%)	Mean (SD): 9.1 (3.9) years
Adult patients				
Abnormal dental examination	Herrou et al. (2022) (33)	62^a^	54 (87.1%)	Mean (SD): 42.2 (14.3) years
Dental abscesses	Baroncelli et al. (2021) (27)^c^	10	2 (20.0%)	Range: 20.1–25.7 years
	Ito et al. (2022) (16)	32	7 (21.9%)	Mean (SD): 40.2 (13.8) years
	Javaid et al. (2022) (15)^b^	127	80 (63.0%)	Range: 18–65 years
		209	167 (79.9%)	
	Skrinar et al. (2019) (12)	232	189 (81.5%)	Mean (SD): 45.6 (12.9) years
	Theodore-Oklota et al. (2018) (42)	18	14 (77.8%)	Mean: 42 years
	Chesher et al. (2018) (13)	59	24 (40.7%)	Median: 37 years
Dental complications	Herrou et al. (2022) (33)	114	78 (68.4%)	Mean (SD): 42.2 (14.3) years
Dental extraction	Chesher et al. (2018) (13)	59	29 (49.2%)	Median: 37 years
Dental implant failure	Ito et al. (2022) (16)	32	3 (9.4%)	Mean (SD): 40.2 (13.8) years
	Skrinar et al. (2019) (12)	232	22 (9.5%)	Mean (SD): 45.6 (12.9) years
Dental involvement	Rafaelsen et al. (2016) (6)	21	9 (42.9%)	Median: 22 years
Excessive cavities	Ito et al. (2022) (16)	32	12 (37.5%)	Mean (SD): 40.2 (13.8) years
	Skrinar et al. (2019) (12)	232	121 (52.2%)	Mean (SD): 45.6 (12.9) years
Hearing impairment	Chesher et al. (2018) (13)	59	8 (13.6%)	Median: 37 years
Hearing loss	Theodore-Oklota et al. (2018) (42)	18	5 (27.8%)	Mean: 42 years
	Skrinar et al. (2019) (12)	232	102 (44.0%)	Mean (SD): 45.6 (12.9) years
Alveolar bone loss^d^	Herrou et al. (2022) (33)	62^a^	44 (71.0%)	Mean (SD): 42.2 (14.3) years
Root canal surgery/endodontic surgery	Berndt et al. (1996) (28	22	3 (13.6%)	Median: 29 years
	Ito et al. (2022) (16)	32	8 (25.0%)	Mean (SD): 40.2 (13.8) years
	Skrinar et al. (2019) (12)	232	166 (71.6%)	Mean (SD): 45.6 (12.9) years
Severe dental disease	Herrou et al. (2022) (33)	62^a^	42 (67.7%)	Mean (SD): 42.2 (14.3) years
Tinnitus	Theodore-Oklota et al. (2018) (42)	18	5 (27.8%)	Mean: 42 years
	Skrinar et al. (2019) (12)	232	106 (45.7%)	Mean (SD): 45.6 (12.9) years

Abbreviations: IQR, interquartile range; SD, standard deviation; XLH, X-linked hypophosphataemia.

^a^ Patients who underwent dental examination.

^b^ Javaid et al. (15), reports outcomes for two groups of patients: subjects from a clinical trial and participants in an online survey. Only the proportion, rather than the absolute number, of patients with each event was reported. Therefore, outcomes are recorded as the range of proportions across the two groups.

^c^ Baroncelli et al. (27), reports incidence during COVID-19 lockdown.

^d^ Includes patients with mild, moderate, and severe alveolar bone loss.

11 publications reported dental issues/diseases and dental interventions. In paediatric patients, a history of dental abscess was reported in 14%–51% of patients (*n* = 148 across three studies (12, 16, 40), excessive cavities were reported in 24%–29% of patients (*n* = 104 across two studies (12, 16), and root canal/endodontic therapy was reported in 0%–17% of patients (*n* = 104 across two studies (12, 16). Among adults, a history of dental abscesses was reported in 20%–82% of patients (*n* = 687 across six studies (12, 13, 15, 16, 27, 42), excessive cavities were reported in 38%–52% of patients (*n* = 264 across two studies (12, 16), and root canal surgery/endodontic therapy was reported in 14%–72% of patients (*n* = 286 across three studies (12, 16, 28).

Hearing problems, which included hearing impairment, hearing loss, and tinnitus, were reported in 8%–9% of paediatric patients (*n* = 90 in one study (12) and 14%–46% of adults (*n* = 309 across three studies (12, 13, 42).

#### 3.2.4 Morbidities associated with conventional therapy

**Figure 5:**
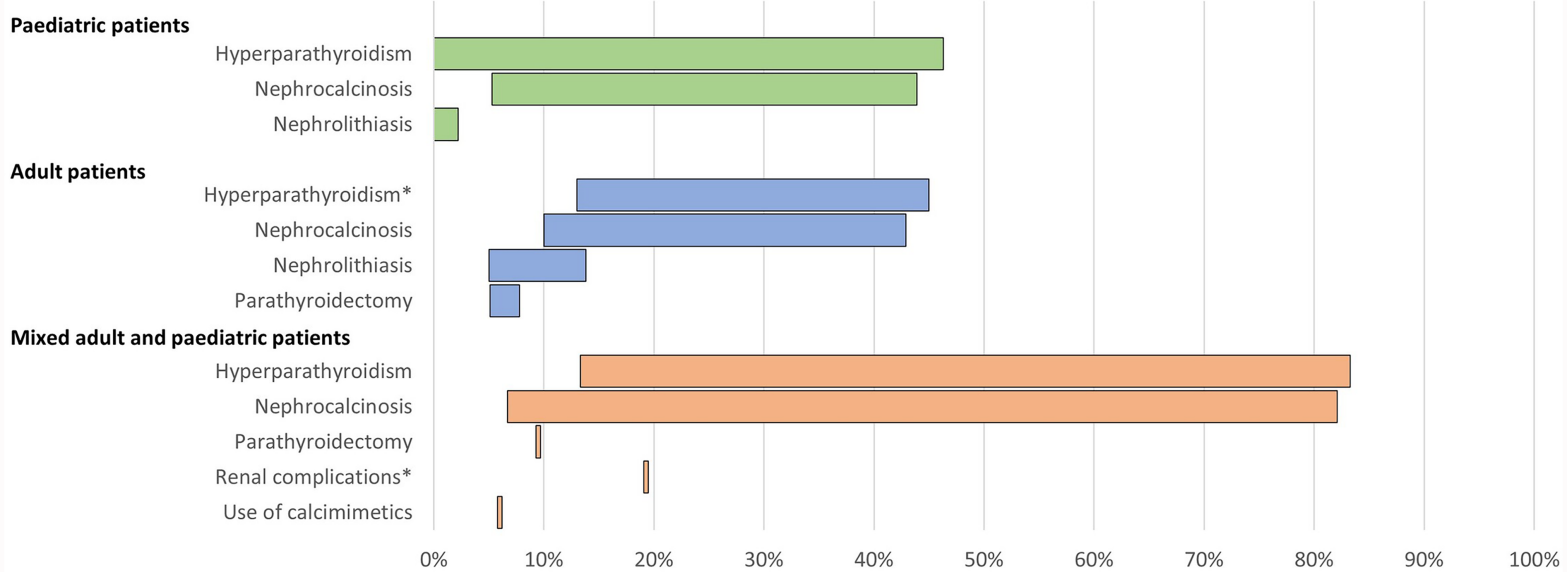
Range of the proportion of patients with a history of morbidities associated with conventional therapy for XLH reported across studies. *Includes Berndt et al. (28), reporting secondary hyperparathyroidism only. Abbreviations: XLH, X-linked hypophosphataemia.

13 publications reported clinical morbidities and healthcare resource use associated with conventional therapy (Table 5; Figure 5).

**Table 5:** Summary of morbidities associated with conventional therapy for XLH.

Outcome	Publication	Sample size (*n*)	Number of patients (%)	Patient age
Paediatric patients				
Hyperparathyroidism	Alon et al. (2003) (26)	41	19 (46.3%)	Range: 10–18 years
	Ito et al. (2022) (16)	14	0 (0.0%)	Mean (SD): 6.8 (3.7) years
	Rodríguez-Rubio et al. (2021) (38)	48	11 (22.9%)	Median (IQR): 2.0 (2.6) years
	Skrinar et al. (2019) (12)	90	16 (17.8%)	Mean (SD): 9.1 (3.9) years
Nephrocalcinosis	Alon et al. (2003) (26)	41	18 (43.9%)	Range: 10–18 years
	Ito et al. (2022) (16)	14	2 (14.3%)	Mean (SD): 6.8 (3.7) years
	Rodríguez-Rubio et al. (2021) (38)	48	8 (16.7%)	Median (IQR): 2.0 (2.6) years
	Rohmiller et al. (1999) (39)	38	2 (5.3%)	Range: 3–213 months
	Skrinar et al. (2019) (12)	90	29 (32.2%)	Mean (SD): 9.1 (3.9) years
Nephrolithiasis	Ito et al. (2022) (16)	14	0 (0.0%)	Mean (SD): 6.8 (3.7) years
	Skrinar et al. (2019) (12)	90	2 (2.2%)	Mean (SD): 9.1 (3.9) years
Adult patients				
Hyperparathyroidism	Ito et al. (2022) (16)	32	5 (15.6%)	Mean (SD): 40.2 (13.8) years
	Jiménez et al. (2021) (35)	20	9 (45.0%)	Mean (SD): 35.6 (13.4) years
	Skrinar et al. (2019) (12)	232	68 (29.3%)	Mean (SD): 45.6 (12.9) years
Hyperparathyroidism (secondary)	Berndt et al. (1996) (28)	23	3 (13.0%)	Median: 29 years
Nephrocalcinosis	Berndt et al. (1996) (28)	23	8 (34.8%)	Median: 29 years
	Chesher et al. (2018) (13)	59	16 (27.1%)	Median: 37 years
	Ito et al. (2022) (16)	32	5 (15.6%)	Mean (SD): 40.2 (13.8) years
	Jiménez et al. (2021) (35)	20	2 (10.0%)	Mean (SD): 35.6 (13.4) years
	Rafaelsen et al. (2016) (6)	21	9 (42.9%)	Median: 22 years
	Skrinar et al. (2019) (12)	232	49 (21.1%)	Mean (SD): 45.6 (12.9) years
Nephrolithiasis	Ito et al. (2022) (16)	32	3 (9.4%)	Mean (SD): 40.2 (13.8) years
	Jiménez et al. (2021) (35)	20	1 (5.0%)	Mean (SD): 35.6 (13.4) years
	Skrinar et al. (2019) (12)	232	32 (13.8%)	Mean (SD): 45.6 (12.9) years
Parathyroidectomy	Chesher et al. (2018) (13)	59	3 (5.1%)	Median: 37 years
	Ito et al. (2022) (16)	32	2 (6.3%)	Mean (SD): 40.2 (13.8) years
	Skrinar et al. (2019) (12)	232	18 (7.8%)	Mean (SD): 45.6 (12.9) years
Mixed adult and paediatric patients				
Hyperparathyroidism	Chung et al. (2002) (29)	15	2 (13.3%)	Median: 5.6 years
	Delacy et al. (2019) (30)	84	70 (83.3%)	Age group: ≥18: *n* = 40, <18: *n* = 44
Nephrocalcinosis	Baroncelli et al. (2021) (27)	26	5 (19.2%)	Range: 3.1–25.7 years
	Chung et al. (2002) (29)	15	1 (6.7%)	Median: 5.6 years
	Delacy et al. (2019) (30)	84	69 (82.1%)	Age group: ≥18: *n* = 40, <18: *n* = 44
Parathyroidectomy	Delacy et al. (2019) (30)	84	8 (9.5%)	Age group: ≥18: *n* = 40, <18: *n* = 44
Renal complications^a^	Moreira et al. (2020) (37)	57	11 (19.3%)	Median: 22 years
Use of calcimimetics	Delacy et al. (2019) (30)	84	5 (6.0%)	Age group: ≥18: *n* = 40, <18: *n* = 44

IQR, interquartile range; SD, standard deviation; XLH, X-linked hypophosphataemia.

^a^ Including nephrocalcinosis, nephrolithiasis, and membranous nephropathy.

In paediatric patients, a history of hyperparathyroidism was reported in 0%–46% of patients (*n* = 193 across four studies (12, 16, 26, 38). Among adults, history of hyperparathyroidism was reported in 13%–45% of patients (*n* = 307 across four studies (12, 16, 28, 35) and parathyroidectomy was reported in 5%–8% of patients (*n* = 323 across three studies (12, 13, 16).

Renal complications were commonplace. In paediatric patients, the prevalence of nephrocalcinosis ranged from 5% to 44% (*n* = 231 across five studies (12, 16, 26, 38, 39), and nephrolithiasis was reported in 0%–2% of paediatric patients (*n* = 104 across two studies (12, 16). In adults, nephrocalcinosis was reported in 10%–43% of patients (*n* = 387 across six studies (6, 12, 13, 16, 28, 35) and nephrolithiasis was reported in 5%–14% of patients (*n* = 284 across three studies (12, 16, 35).

No studies explicitly reported dialysis or other renal replacement therapies.

#### 3.2.5 Productivity

Two publications were identified which reported outcomes related to productivity loss in XLH patients, both of which were interview studies (Table 6). Nevertheless, both studies highlighted the negative impact of XLH on work/school productivity.

**Table 6:** Summary of productivity.

Outcome	Publication	Sample size (*n*)	Number of patients (%)	Patient age
Paediatric patients				
Impact on school attendance	Berndt et al. (1996) (28)^a^	23	7 (30.4%)	Median: 29 years (at last investigation)
Adult patients				
Impact on daily activities: ability to work	Theodore-Oklota et al. (2018) (42)	18	14 (77.8%)	Mean: 42 years

^a^ Study participants had a median age of 29 years but reported impact on productivity during childhood.

In one retrospective evaluation of the clinical course of XLH in German adults (*n* = 23), seven (30%) patients responding to a questionnaire on pain and psychosocial rehabilitation reported missing school repeatedly during childhood due to hospitalisations, leading to repetition of classes and, in four (17%) cases, leading to an inappropriate school qualification (28). In a qualitative study evaluating the patient experience in adults with XLH in the USA (*n* = 18), 14 (78%) patients reported an impact of XLH on their ability to work (42).

## 4 Discussion

The findings of this structured literature review suggest that XLH is associated with a substantial economic burden in both paediatric and adult patient populations across multiple categories, including the use of pharmacological therapy, orthopaedic interventions, treatment of morbidities related to low phosphate levels and those caused by conventional pharmacological therapy, and productivity.

In particular, a high proportion of patients with XLH sustain fractures and require corrective orthopaedic procedures, both of which are associated with substantial healthcare resource use (43–45). Evidence also suggests that orthopaedic surgery in people with XLH may be associated with a particularly high economic burden. For example, orthopaedic procedures are associated with a high prevalence of complications in XLH patients, with an average of one complication per surgical procedure (46). Furthermore, the treatment goal of surgery was found not to have been achieved in 28% of procedures, and half of these resulted in permanent sequalae or new pathology. Ongoing surgeries also negatively impact the lives of people with XLH, i.e., there is a substantial patient burden associated with frequent, often painful surgeries that can be distressing and disruptive to education and work (18, 47).

The review also shows that resource use is generally higher in adults compared with paediatric patients, with the exceptions of pharmacological therapy (which is typically only recommended in symptomatic adults (14, 19) and guided growth/growth plate stapling (the use of which has increased in recent years as a means of avoiding osteotomy (14, 34). This finding is consistent with the natural history of XLH as a progressive lifelong condition, during which morbidities accumulate over time as patients age. This suggests that earlier diagnosis along with more effective treatment and management of XLH in childhood may help to reduce or avoid subsequent healthcare resource use and costs in later life (12, 13). This is supported by the opinions of a working group with expertise in the biology and management of XLH, who indicated that adequate treatment of XLH in childhood should substantially improve lifetime prognosis, due to improved skeletal development which should prevent or reduce the occurrence of or need for correction of skeletal deformities (2).

A number of published studies have shown the high overall burden of disease associated with XLH (12, 13, 15, 16). The present study is, to the authors' knowledge, the first to collate data on the healthcare resource use and productivity burden associated with the disease. Findings emphasise the lifelong impact of the condition, highlight its complexity, and suggest that effective treatments are necessary to reduce the clinical and economic burden of XLH. A previous systematic review collated evidence on the burden of disease in adults with XLH, taking a broad perspective and extracting information on clinical manifestations, symptoms, and humanistic and socioeconomic burden of disease (48). However, this review considered economic burden in the strict financial sense, and therefore did not report any findings within this category, whereas our review considered any morbidities, events, or procedures with healthcare resource use implications, as well as the productivity burden of disease. Additionally, our review included outcomes for paediatric patients with XLH, as well as adults, therefore characterising the progressive burden of disease as patients age.

Our study has a number of limitations. First, in most cases, the identified studies reported cumulative incidences of events and procedures associated with resource use; age at which events occurred was seldom reported. Based on this information, it was not possible to determine the rate of event occurrence (for example, the mean number of fractures or orthopaedic procedures per year). A small number of studies provided information from which timing of events could be inferred. Javaid et al., reported prevalence of musculoskeletal features of XLH stratified by age group, showing a substantial prevalence of fractures even in the youngest adult age group (18–29 years), in addition to osteoarthritis and osteophytes, indicating that adult morbidities of XLH associated with HCRU can develop at a relatively young age (15). This is supported by findings from Skrinar et al., and Ito et al., which reported an age of first fracture of between 26.4 and 29.7 years, depending on fracture location (12, 16). Additionally, Fucentese et al., reported a median age at first corrective surgery of 7.8 years, indicating a requirement for corrective procedures from a young age (31).

Similarly, few studies reported the duration of treatment with conventional therapy, or the relationship between duration of treatment and associated complications. Rafaelsen et al., reported that all children who developed nephrocalcinosis did so within 5 years of treatment with oral phosphate and active vitamin D, and started treatment earlier on average than those who did not develop nephrocalcinosis (6). Additionally, the study reported a trend towards a higher daily dose of oral phosphate (in terms of mg/kg) in those developing nephrocalcinosis. However, the sparsity of supporting information indicates that further research in this area is required.

Second, publications from a variety of countries were included in the review, with differing healthcare systems, access to treatment, and patient populations, which complicates aggregating results and cross-study comparisons. This may explain the wide range of values for some outcomes. For example, a study conducted in Japanese and Korean patients reported relatively low levels of morbidities associated with XLH and conventional therapy, which may indicate that disease management in these countries differs from that of the settings of other publications included in the review (16). Although reported outcomes were stratified by age group (paediatric vs. adult) wherever possible, studies showed variation in average age within these groups. Since the burden of XLH accumulates over time, this is likely to result in variation in estimates of the cumulative incidence of morbidities, events, and procedures.

Third, in some cases, direct estimates of resource use were not available, only indirectly through the prevalence of clinical events, such as fractures or hyperparathyroidism. Such events can be complicated to treat and, as such, it was not possible to precisely quantify specific resource implications, particularly considering the evidence that procedures may be especially burdensome in XLH patients (46). Furthermore, not all resource use associated with XLH may be captured in the published literature. For example, clinical guidelines recommend that XLH patients are managed by multidisciplinary teams, with routine blood testing, twice-yearly dentist visits, and kidney ultrasonography carried out at least every two years in patients receiving pharmacological treatment (14, 19). However, no empirical evidence of such resource use was reported in the identified publications.

Fourth, to maximise available evidence, this review did not restrict included studies based on diagnostic criteria for XLH. In the majority of studies, XLH was diagnosed on the basis of a *PHEX* mutation and/or a combination of clinical and biochemical features. However, the possibility that some patients included in these studies were misdiagnosed with XLH cannot be excluded.

Fifth, a potential source of bias in the study may be that the identified publications only included patients who are users of healthcare services, owing to the way in which patients were recruited. However, considering the substantial resource burden of XLH across a number of clinical areas, it is likely that most patients would have been users of healthcare services.

Finally, research into XLH is rapidly progressing, as demonstrated by the relative recency of articles included in this review; the majority were published within 5 years of the search date. Therefore, additional evidence regarding the healthcare resource use and productivity burden of XLH published after the search date of the current review may be available at the time of publication. This highlights the need for ongoing work to summarize the evolving evidence base in XLH.

Owing to its relatively recent availability, burosumab was not included in the review. However, it is plausible that burosumab could impact resource use across several categories. For example, clinical trial evidence shows that burosumab produces significant improvements in rickets, which could reduce the requirement for orthopaedic surgery later in life (49). Clinical trials additionally show that burosumab produces a significant improvement in serum phosphate levels (vs. conventional therapy in paediatric patients and vs. placebo in adults), which could reduce incidence of morbidities due to chronic hypophosphatemia (25, 49–51). Furthermore, the summary of product characteristics for burosumab specifies that treatment with oral phosphate and active vitamin D should be discontinued prior to initiation of burosumab (52), which is likely to reduce the incidence of new morbidities related to conventional therapy (for example, nephrocalcinosis, kidney stones, hyperparathyroidism, and impaired renal function). These points are backed up by findings from a recent elicitation exercise in which experts showed a high level of agreement that burosumab is likely to reduce the incidence of fractures and morbidities associated with conventional therapy (53).

Further research in this area would be beneficial. First, in quantifying the timing of morbidities and procedures leading to healthcare resource use over time. Findings of this literature review highlight the progressive burden of XLH, but few of the included studies reported the age at which events occurred. Second, the majority of evidence identified in this literature review relates to the burden on healthcare systems; further research quantifying the economic burden of XLH on a societal basis would be helpful. For instance, published evidence indicates a substantial burden on caregivers of patients with XLH, although this impact was not quantified (54). Finally, it is evident that further research into the impact of XLH, its associated morbidities and interventions, on loss of productivity at school and work is important.

In conclusion, the results of this structured literature review emphasise the lifelong impact of XLH, showing that the disease is associated with a substantial economic burden across many areas, including pharmacological treatment, management of pain and mobility, orthopaedic procedures, morbidities due to XLH or conventional therapy, and productivity loss.
