# Supplementary material for: A literature review of the healthcare resource use and productivity burden of X-linked hypophosphataemia
Source: Front Health Serv. 2025 Apr 9;5:1285246. doi: 10.3389/frhs.2025.1285246 (PMC12014534; doi:10.3389/frhs.2025.1285246)
Supplement: Supplementary file 1 [file Supplementaryfile1.pdf]

Supplementary Material

1 Supplementary File 1: literature search strategies

Table 1. Ovid SP® search strategy for Embase® (run on 16 August 2022)

# ▲	Category	Searches	Results
1.	Population	exp X linked hypophosphatemic rickets/	1,024
2.		exp familial hypophosphatemic rickets/	1,373
3.		((X linked or familial or hereditary) adj3 (hypophosphat?emi* or rickets or osteomalacia)).tw.	1,884
4.		XLH.tw.	921
5.		(hypophosphat?emic adj3 rickets).tw.	1,776
6.		Or/1-5	3,042
7.	Resource use	health economics/	34,549
8.		cost of illness/	20,688
9.		cost control/	73,627
10.		disease burden/	35,818
11.		medical leave/	8,027
12.		healthcare financing/	13,725
13.		work disability/	5,561
14.		absenteeism/	18,971
15.		presenteeism/	1,995
16.		productivity/	45,864
17.		caregiver/	101,368
18.		length of stay/	240,922
19.		hospitalization cost/	8,933
20.		health care utilization/	85,968
21.		employment/	72,569
22.		echography/	360,548
23.		radiography/	199,501

# ▲	Category	Searches	Results
24.		((economic or societ* or socioeconomic or socio economic or illness or disease or patient* or caregiver* or carer* or productiv* or employ*) adj3 (burden or impact or consequence?)).tw.	247,817
25.		(productivity or cost* or price* or pricing or pharmacoeconomic* or pharmaco-economic* or expenditure* or expens* or visit* or financ* or inpatient or outpatient or hospitali* or (length adj5 stay)).tw.	2,486,922
26.		(resource* adj2 ("use" or usage or utili* or allocat*)).tw.	73,277
27.		(leave adj2 (medical or sick or disability)).tw.	7,958
28.		(echo* or ultrason* or radio* or x ray or ro?ntgen*).tw.	2,429,437
29.		or/7-28	5,552,602
30.		6 and 29	682
31.	Clinical guidelines	practice guideline/	508,042
32.		disease management/	20,508
33.		monitoring/	171,408
34.		((clinical or treatment? or therap* or surg* or ortho*) adj2 (guideline? or guidance or recommend* or monitor* or surveillance or practic* or procedur* or manage*)).tw.	1,008,436
35.		Or/31-34	1,588,926
36.		6 and 35	160
37.	Intervention rates	incidence/	512,745
38.		prevalence/	863,609
39.		epidemiology/	233,813
40.		osteotomy/	36,431
41.		epiphysis plate/	2,672
42.		hip replacement/	5,419
43.		knee replacement/	6,075
44.		hip arthroplasty/	19,338
45.		knee arthroplasty/	16,980
46.		spine surgery/	29,773

# ▲	Category	Searches	Results
47.		laminectomy/	24,043
48.		spinal cord decompression/	7,476
49.		discectomy/	5,503
50.		parathyroidectomy/	12,252
51.		tooth extraction/	25,280
52.		tooth root canal/	4,844
53.		dental pulp capping/	509
54.		tooth implant/	16,957
55.		calcimimetic agent/	1,020
56.		burosumab/	344
57.		vitamin d/	89,581
58.		phosphate/	86,882
59.		analgesia/	142,993
60.		walking aid/	5,212
61.		hearing aid/	14,106
62.		(osteotom* or plate? or ((replace* or arthroplasty) adj2 (hip or knee)) or laminectomy or discectomy or parathyroidectomy or ((spin* or invertebra* or disc*) adj2 (decompress* or fusion? or surger* or operat*))).tw.	426,557
63.		((tooth or dental) adj2 (extract* or canal or cap* or implant* or remov* or resect* or surger* or procedure*)).tw.	39,118
64.		(burosumab or crysvita or KRN 23 or KRN23).tw.	324
65.		(pharmacologic* or (oral* adj2 phosphate) or vitamin d or calcimimetic?).tw.	566,936
66.		((pain or analgesi*) adj2 (medic* or "use" or usage or utili* or prescri* or consum*)).tw.	45,817
67.		((walk* or ambulat* or hear*) adj2 (device? or aid*)).tw.	19,934
68.		(incidence* or prevalence* or rate* or epidemiolog* or clinical or manifest* or complicat* or comorbidit* or morbidit*).tw.	11,848,388
69.		Or/37-39	1,520,982
70.		Or/40-61	523,017

# ▲	Category	Searches	Results
71.		69 and 70	28,516
72.		Or/62-67	1,083,777
73.		72 and 68	472,839
74.		6 and (71 or 73)	757
75.	Complication rates	incidence/	512,745
76.		prevalence/	863,609
77.		epidemiology/	233,813
78.		nephrolithiasis/	38,300
79.		fracture/	92,547
80.		stress fracture/	7,148
81.		pseudoarthrosis/	11,937
82.		fracture nonunion/	15,770
83.		tooth abscess/	1,245
84.		periodontitis/	29,779
85.		hearing impairment/	65,920
86.		tinnitus/	23,278
87.		kidney calcification/	5,722
88.		hyperparathyroidism/	18,208
89.		kidney function/	154,661
90.		vertebral canal stenosis/	11,856
91.		osteoarthritis/	96,083
92.		short stature/	16,491
93.		bone deformation/	1,636
94.		bone malformation/	6,656
95.		arthralgia/	72,036
96.		bone pain/	18,329
97.		(fracture or pseudofracture* or pseud?arthros#s or dental or abscess or tinnitus or deaf* or nephrolithiasis or hyperparathyroidism or deformit* or malalign* or malform* or ((kidney or renal) adj2 (stone? or calcul* or impair* or function? or failure? or problem*)) or stenosis or osteo* or short or calcificat* or (calcium adj2	3,297,129

# ▲	Category	Searches	Results
		deposit?) or (hear* adj2 (loss or impair* or problem* or failure?)) or ((join or bone) adj2 pain)).tw.	
98.		(incidence* or prevalence* or rate* or epidemiolog* or clinical or manifest* or complicat* or comorbidit* or morbidit*).tw.	11,848,388
99.		Or/75-77	1,520,982
100.		Or/78-96	646,983
101.		99 and 100	47,713
102.		97 and 98	1,623,019
103.		6 and (101 or 102)	1,013
104.	Total	30 or 36 or 74 or 103	1,510
105.	Total after time	limit 104 to yr="1992 -Current"	1,371
106.	Total after limiting to conference from the past 2/3 years	exp conference publication/ or conference abstract/ or (conference adj (abstract or publication or review or proceeding)).pt.	5,291,191
107.		limit 106 to yr="1992 - 2019"	4,492,903
108.		105 not 107	975
109.	Total after language limit	Limit 108 to English language	903
110.	Total after publication type and human limits	(exp animal/ or exp invertebrate/ or nonhuman/ or animal experiment/ or animal tissue/ or animal model/ or exp plant/ or exp fungus/) not (exp human/ or human tissue/)	7,416,233
111.		109 not 110	840
112.		exp case study/ or exp case report/ or exp letter/ or exp editorial/ or exp preliminary communication/ or exp note/	5,306,276
113.		111 not 112	596

Table 2. Ovid SP® search strategy for Medline® (run on 16 August 2022)

# ▲	Category	Searches	Results
1.	Population	exp rickets, hypophosphatemic/	851
2.		familial hypophosphatemic rickets/	744

# ▲	Category	Searches	Results
3.		(X linked or familial or hereditary) adj3 (hypophosphat?emi* or rickets or osteomalacia)).tw.	1,385
4.		XLH.tw.	535
5.		(hypophosphat?emic adj3 rickets).tw.	1,314
6.		or/1-5	2,225
7.	Resource use	health care economics and organizations/	0
8.		cost of illness/	30,893
9.		cost control/	21,651
10.		sick leave/	6,570
11.		healthcare financing/	1,201
12.		work capacity evaluation/	6,245
13.		absenteeism/	9,669
14.		presenteeism/	539
15.		efficiency/	15,045
16.		caregivers/	46,742
17.		length of stay/	100,226
18.		hospitalization/	129,456
19.		health care costs/	43,428
20.		health resources/	14,604
21.		employment/	49,401
22.		ultrasonography/	196,190
23.		radiography/	325,234
24.		((economic or societ* or socioeconomic or socio economic or illness or disease or patient* or caregiver* or carer* or productiv* or employ*) adj3 (burden or impact or consequence?)).tw.	157,957
25.		(productivity or cost* or price* or pricing or pharmacoeconomic* or pharmaco-economic* or expenditure* or expens* or visit* or financ* or inpatient or outpatient or hospitali* or (length adj5 stay)).tw.	1,735,870

# ▲	Category	Searches	Results
26.		(resource* adj2 ("use" or usage or utili* or allocat*)).tw.	49,511
27.		(leave adj2 (medical or sick or disability)).tw.	6,204
28.		(echo* or ultrason* or radio* or x ray or ro?ntgen*).tw.	1,878,066
29.		((pain or analgesi*) adj2 (medic* or "use" or usage or utili* or prescri* or consum*)).tw.	28,161
30.		or/7-29	4,165,477
31.		6 and 30	446
32.	Clinical guidelines	practice guideline/	29,968
33.		disease management/	42,869
34.		drug monitoring/	23,233
35.		((clinical or treatment? or therap* or surg* or ortho*) adj2 (guideline? or guidance or recommend* or monitor* or surveillance or practic* or procedur* or manage*)).tw.	702,258
36.		or/32-35	776,140
37.		6 and 36	83
38.	Intervention rates	incidence/	294,855
39.		prevalence/	333,666
40.		epidemiology/	12,559
41.		osteotomy/	32,472
42.		growth plate/	5,076
43.		arthroplasty, replacement, hip/	32,706
44.		arthroplasty, replacement, knee/	29,683
45.		spinal fusion/	29,942
46.		laminectomy/	10,365
47.		decompression, surgical/	17,488
48.		diskectomy/	5,818
49.		parathyroidectomy/	5,861
50.		tooth extraction/	20,304
51.		root canal therapy/	13,625

# ▲	Category	Searches	Results
52.		dental pulp capping/	2,158
53.		dental implants/	25,317
54.		calcimimetic agents/	426
55.		vitamin d/	40,806
56.		phosphates/	65,930
57.		pain management/	39,641
58.		walking/	39,219
59.		hearing aids/	9,668
60.		antibodies, monoclonal, humanized/	51,565
61.		(osteotom* or plate? or ((replace* or arthroplasty) adj2 (hip or knee)) or laminectomy or discectomy or parthyroidectomy or ((spin* or invertebra* or disc*) adj2 (decompress* or fusion? or surger* or operat*))).tw.	336,990
62.		((tooth or dental) adj2 (extract* or canal or cap* or implant* or remov* or resect* or surger* or procedure*)).tw.	37,549
63.		(burosumab or crysvita or KRN 23 or KRN23).tw.	134
64.		(pharmacologic* or (oral* adj2 phosphate) or vitamin d or calcimimetic?).tw.	413,136
65.		((pain or analgesi*) adj2 (medic* or "use" or usage or utili* or prescri* or consum*)).tw.	28,161
66.		((walk* or ambulat* or hear*) adj2 (device? or aid*)).tw.	16,010
67.		(incidence* or prevalence* or rate* or epidemiolog* or clinical or manifest* or complicat* or comorbidit* or morbidit*).tw.	8,780,044
68.		Or/38-40	613,510
69.		Or/41-60	452,420
70.		68 and 69	10,148
71.		Or/61-66	823,303
72.		71 and 67	331,626

# ▲	Category	Searches	Results
73.		6 and (70 or 72)	426
74.	Complication rates	incidence/	294,855
75.		prevalence/	333,666
76.		epidemiology/	12,559
77.		kidney calculi/	20,590
78.		fracture dislocation/	514
79.		fractures, stress/	3,718
80.		fractures, ununited/	6,296
81.		pseudarthrosis/	5,433
82.		periodontal abscess/	633
83.		periodontitis/	20,118
84.		hearing loss/	19,058
85.		deafness/	28,060
86.		tinnitus/	9,059
87.		nephrocalcinosis/	2,188
88.		hyperparathyroidism/	13,612
89.		kidney failure, chronic/	99,337
90.		spinal stenosis/	6,938
91.		osteoarthritis/	41,764
92.		bone malalignment/	1,843
93.		body height/	37,862
94.		arthralgia/	9,539
95.		(fracture? or pseudofracture* or pseud?arthros#s or dental or abscess or tinnitus or deaf* or nephrolithiasis or hyperparathyroidism or deformit* or malalign* or malform* or ((kidney or renal) adj2 (stone? or calcul* or impair* or function? or failure? or problem*)) or stenosis or osteo* or short or calcificat* or (calcium adj2 deposit?) or (hear* adj2 (loss or impair* or problem* or failure?)) or ((join or bone) adj2 pain)).tw.	2,627,831
96.		(incidence* or prevalence* or rate* or epidemiolog* or clinical or manifest* or	8,780,044

# ▲	Category	Searches	Results
		complicat* or comorbidit* or morbidit*).tw.	
97.		Or/74-76	613,510
98.		Or/77-94	316,538
99.		97 and 98	13,410
100.		95 and 96	1,128,578
101.		6 and (99 or 100)	591
102.	Total	31 or 37 or 73 or 101	951
103.	Total after time	limit 102 to yr="1992 -Current"	809
104.	Total after language limit	limit 103 to English language	753
105.	Total after publication type and human limits	exp case reports/ or exp letter/ or exp editorial/ or exp news/	4,078,633
106.		104 not 105	544
107.		(exp animals/ or exp invertebrates/ or exp animal experimentation/ or exp models, animal/ or exp plants/ or exp fungi/) not exp humans/	5,454,078
108.		106 not 107	500

Table 3. Ovid SP® search strategy for EconLit (run on 16 August 2022)

# ▲	Category	Searches	Results
1.	Population	((X linked or familial or hereditary) adj3 (hypophosphat?emi* or rickets or osteomalacia)).tw.	0
2.		XLH.tw.	0
3.		(hypophosphat?emic adj3 rickets).tw.	0
4.		or/1-3	0
