# Supplementary material for: A literature review of the healthcare resource use and productivity burden of X-linked hypophosphataemia
Source: Front Health Serv. 2025 Apr 9;5:1285246. doi: 10.3389/frhs.2025.1285246 (PMC12014534; doi:10.3389/frhs.2025.1285246)
Supplement: Supplementary file 2 [file Supplementaryfile2.pdf]

Supplementary Material

1 Supplementary File 2: literature search inclusion and exclusion criteria

Table 1. Inclusion and exclusion criteria for publications identified in the literature searches

**PICOTS topic**	**Inclusion criteria**	**Exclusion criteria**
Population	• All patients with XLH (adult and paediatric)	• Non-XLH<br>• Sample size < 10 patients
Interventions and comparators	• Any	• Not applicable
Outcomes	• Healthcare resource use and productivity impact of XLH, including but not limited to*:<br>○ Incidence and proportion of patients experiencing events incurring resource use<br>○ Number of hospitalisations<br>○ Length of hospital stay<br>○ Number of visits (e.g., outpatient, emergency, physician, etc.)<br>○ Medicine utilisation<br>○ Productivity loss (e.g., presenteeism, absenteeism)<br>○ Caregiver burden<br>○ Hospital readmission rate	• Articles reporting only cost data
Study design	• Observational studies<br>• Database claims studies<br>• Prospective studies<br>• Retrospective studies<br>• Systematic reviews/economic evaluations	• Editorials<br>• News articles<br>• Non-systematic reviews<br>• Commentaries<br>• Articles making only general reference to resource use (not based on data)
Time	• 1 January 1992 to 16 August 2022	• Pre-1992
Language	• English	• Non-English

* Morbidities related to low phosphate levels and conventional therapy were also captured; while these are not traditional resource use categories, in context of this review they have

resource use implications.

Abbreviations: PICOTS, population, intervention, comparison, outcome, time, study; XLH, X-linked hypophosphataemia.
